# Differential Effects of Low-Intensity Pulsed Ultrasound and Antifungals on *Candida albicans* and *Candida glabrata*: Implications for Drug Efficacy

**DOI:** 10.3390/jof12060399

**Published:** 2026-05-30

**Authors:** Sichen Liu, James Townley, Amir Seyedmousavi, Joseph A. Frank

**Affiliations:** 1 Frank Laboratory, Radiology and Imaging Sciences, Clinical Center, National Institutes of Health, Bethesda, MD 20892, USA; 2 Laboratory of Clinical Microbiology and Immunology, National Institute of Allergy and Infectious Diseases, National Institutes of Health, Bethesda, MD 20892, USA; 3 Alice L. Walton School of Medicine, Bentonville, AR 72712, USA; 4 Microbiology Service, Department of Laboratory Medicine, Clinical Center, National Institutes of Health, Bethesda, MD 20892, USA; 5 National Institute of Biomedical Imaging and Bioengineering, National Institutes of Health, Bethesda, MD 20892, USA

**Keywords:** low-intensity pulsed ultrasound, LIPUS, *Candida albicans*, *Candida glabrata*, sub-MIC antifungal therapy, adenylate kinase assay

## Abstract

Invasive fungal infections pose significant clinical challenges, owing to limited antifungal efficacy and poor tissue drug penetration. This study investigated whether low-intensity pulsed ultrasound (LIPUS) could enhance the antifungal activity of sub-minimal inhibitory concentrations (sub-MICs) of amphotericin B (AmB) and micafungin (MFG) against two strains from two phylogenetically distinct *Candida* species: *Candida albicans* and *Candida glabrata*. Growth inhibition was assessed following LIPUS (15 min, 50% duty cycle, 1 W/cm^2^) alone or in combination with sub-MIC antifungals. Time-kill assays and adenylate kinase (AK) release evaluated the cell viability and membrane integrity, respectively. LIPUS transiently but markedly delayed *C. albicans* growth and enhanced the antifungal effects of both AmB and MFG at sub-MIC levels. The combination of LIPUS and ¼ MIC AmB reduced CFU counts by over 3 log at 12 h and induced higher AK release compared to controls, indicating membrane leakage. In contrast, *Candida glabrata* showed minimal susceptibility to LIPUS, with low AK release and mitigation of the fungistatic effects of sub-MIC MFG. Our study demonstrates that LIPUS can potentiate sub-MIC antifungals against *C. albicans* but may have the opposite effect in *C. glabrata*. This strain-specific variation in response to LIPUS underscores the need for further investigation before LIPUS can be considered as a treatment-enhancement strategy.

## 1. Introduction

Recent advancements in therapies for oncological and rheumatological diseases have significantly improved patient outcomes, but these treatments have also led to a growing population of immunocompromised individuals who are increasingly susceptible to opportunistic infections, including invasive fungal diseases. One such infection, candidiasis, is caused by an overgrowth of *Candida* species and is the second most common invasive fungal disease in patients undergoing stem cell transplantation [1]. Candidiasis may present as thrush in Acquired Immunodeficiency Syndrome (AIDS) or cancer treated with chemotherapy [2]. In immunocompetent patients, *Candida* spp. often colonize catheters leading to catheter-related infections [3]. Candidiasis typically includes all *Candida* species, with *C. albicans* representing most infections and *C. glabrata* accounting for up to 18% of systemic infections [4]. *Candida glabrata* has recently been renamed to *Nakaseomyces glabratus* due to its genetic divergence [5]. Given the recency of this taxonomic revision, we will continue to use the former nomenclature in this work.

These two *Candida* species are known to exhibit distinct responses to environmental stressors such as heat, low pH, and oxidants. *C. glabrata* has high thermotolerance up to the 47–50 °C range [6]. This species also displays unique temperature-dependent responses to antifungals. For instance, *C. glabrata* shows greater tolerance to azoles at 37 °C than at 25 °C; an opposite trend was observed in *C. albicans* [7]. Furthermore, *C. glabrata* demonstrates remarkable versatility in its rapid adaptation to echinocandin-class antifungals, such as micafungin, often through mutations in the *FKS1* gene [8]. Lastly, *C. glabrata* has a remarkable resilience against oxidative stress, not seen in *C. albicans* [9]. This pronounced adaptability makes *C. glabrata* a good comparison to *C. albicans*.

Systemic antifungals currently approved for the treatment of invasive fungal infections (IFIs) include echinocandins, azoles, and polyenes [10], each of which exerts its effects through a distinct mechanism of action. Amphotericin B (AmB) binds to ergosterol, the main component of fungal membrane sterols, and forms large pores that disrupt cell function [11]. In contrast, echinocandins such as micafungin (MFG) directly target the fungal cell wall by inhibiting beta-(1,3)-D-glucan synthase [12]. However, antifungal medications can carry substantial side effects, and treatments can be interrupted due to the patient’s inability to tolerate the medications [13,14].

Therapeutic Ultrasound (TUS) is a noninvasive treatment modality using mechanical waves at frequencies greater than 20 KHz and can serve as an adjunct to current antifungal therapy [15]. High-intensity TUS is used for ablation purposes, while low-intensity pulsed ultrasound (LIPUS), defined as pulsed exposure with an intensity < 3 Watt/cm^2^ (W·cm^−2^), has been used in the clinic to enhance musculoskeletal and soft-tissue healing, particularly at 1 MHz [16,17]. We chose our ultrasound frequency based on safety and expected biological effects. Ultrasound near 1 MHz has been evaluated in multiple clinical trials and has a well-established safety profile, whereas lower frequencies (below ~100 kHz) have been used far less in patients and therefore have less safety data [18]. Lower-frequency ultrasound is also more likely to trigger cavitation, in which microscopic gas bubbles (microbubbles, MBs) form and collapse in the surrounding fluid, generating intense, localized mechanical forces that can damage cells. By comparison, higher-frequency ultrasound (≥1 MHz) tends to produce more heating [18,19]. If not carefully controlled, both cavitation and heating can cause tissue injury. We selected a 1 MHz LIPUS regimen to leverage a clinically familiar and better-characterized operating range.

Beyond these effects, ultrasound has also demonstrated in vitro antimicrobial activity, particularly against bacteria at frequencies < 50 kHz [15]. There have been several proposed mechanisms. With respect to antimicrobial effects, lower-frequency ultrasound generates MBs and therefore increases the amount of cavitation [20]. When these MBs collapse, microjets and acoustic streams form as physical consequences, and can physically disrupt cell membranes and walls of bacteria [21]. However, effects on cells are not always cavitation-driven. In the absence of detectable cavitation, Krasovitski et al. demonstrated that the push-pull oscillating acoustic pressure can lead to cellular membrane bilayer disruption [22]; two layers are pulled apart when the acoustic negative pressure overcomes the molecular attractive forces between the two membrane leaflets and then pushed back together by the positive pressure. Consistent with this model, LIPUS without cavitation has been reported to elicit biological effects in mammalian cells, including human keratinocytes [23].

Indeed, in vitro antimicrobial effects from ultrasound have been reported most consistently at lower frequencies (<50 kHz) where cavitation is more readily induced [15]. In particular, LIPUS at frequencies below 50 kHz has been used in vitro against *C. albicans* with AmB and AmB-containing nanoparticles in both planktonic and biofilm settings [24,25]. Previous studies have shown there is synergy when combining LIPUS with antifungals such as AmB or Fluconazole at concentrations much higher than the minimal inhibitory concentration (MIC) [24,26]. However, these studies did not follow the fungicidal dynamics over 24 h. To our knowledge, the effects of LIPUS on other key *C. albicans* virulence factors, such as hyphae formation, have not yet been investigated. Furthermore, the impact of LIPUS on *C. glabrata* remains entirely unexplored.

Additionally, evaluating for potentially additive or synergistic effects below 1× MIC should be warranted, because infected tissues often exhibit very poor drug penetration. Achieving antifungal concentrations exceeding 1× MIC at the site of infection is frequently unrealistic. For example, infected lung tissue has been reported to contain only 16% of the AmB concentration observed in healthy, uninfected tissue, while another study demonstrated low MFG penetration in murine hepatic *Candida* abscesses [27,28]. Thus, it is critical to explore fungal microbiological response in the presence of sub-MIC drug levels in combination with LIPUS to determine if the combination will improve local drug efficacy.

In this study, we investigated if LIPUS can enhance the efficacy of antifungal agents at lower drug concentrations in an in vitro model involving kinetic growth and time-kill assays of *C. albicans* and *C. glabrata,* two phenotypically and biologically distinct *Candida* species. As proof of concept, our findings suggest that the ability of LIPUS to enhance in vitro antifungal activity varies substantially between two strains from two species. Specifically, for *C. albicans*, there appears to be a transient potential for combining LIPUS with sub-MIC antifungal concentrations, whereas *C. glabrata*, sonication in the presence of sub-MIC antifungal levels did not confer benefit and may be unfavorable under these conditions.

## 2. Materials and Methods

### 2.1. Fungal Strains

The *C. albicans* strain ATCC 10231 and *C. glabrata* strain ATCC 15126 were used in this study. These organisms were cultured on Sabaroud dextrose (SD) agar, with plates stored at 4 °C and refreshed every two weeks.

### 2.2. Antifungal Drugs

Amphotericin B (AmB) and micafungin (MFG) were obtained from Sigma (St. Louis, MO, USA) and diluted in DMSO as a solvent. Powdered antifungal was dissolved in DMSO at room temperature, then vortexed until complete solvation. The solutions were aliquoted and stored at −80 °C for single-use applications. Prior to each experiment, drug aliquots were diluted in Roswell Park Memorial Institute (RPMI) media with 3-(N-morpholino) propanesulfonic acid (MOPS) (Sigma, St. Louis, MO, USA) broth at a pH of 7 following CLSI M27 guidelines [29].

### 2.3. Antifungal Susceptibility Testing

The susceptibility profiles for *C. albicans* and *C. glabrata* isolates were determined using broth microdilution methods using CLSI M27 ED4, 2017 guidelines [29]. Briefly, fresh yeast colonies were grown on SD agar 24 h prior. One colony was resuspended in 5 mL of sterile water and suspension density was adjusted to 0.5 McFarland (McF). Dictated by guidelines, 20 uL of *C. albicans* and 15 uL of *C. glabrata* suspension were added to respective 11 mL of RPMI broth, then 100 uL of suspension was placed into each well of MBL-101 antifungal plate (Match Biolab, Rockville, MD, USA) for a total of 200 uL and MIC was read after incubation for 24 h at 37 °C with the MIC endpoint defined as the lowest concentration that causes 90 to 100% growth inhibition relative to the drug-free growth control. Sterility control wells were included. The experiment was performed in triplicate. 

### 2.4. Low-Intensity Pulsed Ultrasound (LIPUS)

**Figure 1:**
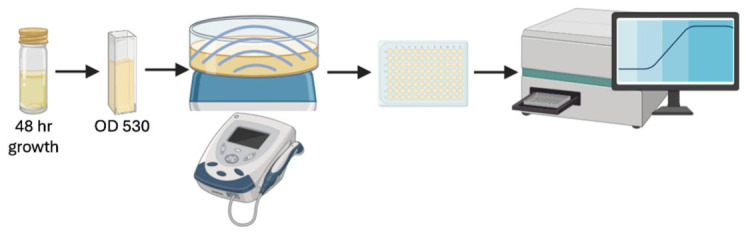
LIPUS experimental setup for growth inhibition assays. Arrows represent procedural next-step.

The LIPUS experiments were performed using Mettler Sonicator 740 (Mettler Electronics, Anaheim, CA, USA) utilizing a 5 cm^2^ linear dual frequency transducer at 1 MHz with a pressure of 1 W·cm^−2^, 50% duty cycle, pulse repetition frequency of 100 kHz, and US burst of 5 ms with treatment duration varying from 1 to 15 min. For LIPUS treatment, transducers were set facing up and leveled (Figure 1). A 35 mm Nunclon Delta petri dish (Corning Inc., Corning, NY, USA) was placed on top with Aquasonics 100 coupling gel (Parker Laboratories Inc, Fairfield, NJ, USA) between the transducer and the bottom of the dish. Non-LIPUS treatment groups were incubated at 37 °C for the sonication duration of their counterparts. Each treatment was performed with two calibrated Mettler systems serving as duplicates for the experiments. Mettler Sonicator 740 was calibrated using a force balance Ohmic UPM-DT-50SP (Ohmic Instruments, St. Charles, MO, USA) every 3 months (Supplementary Figure S1). For LIPUS studies, stable and inertial cavitation of media was determined using Onda MCT-2000 cavitation meter with HCT-0320 probe (Ondasonics, Sunnyvale, CA, USA). LIPUS setup was the same as treatment studies, with transducers set facing up with a 35 mm petri dish placed on top. Coupling gel was placed between the transducer and the bottom of the dish, and the dish was filled with degassed water, RPMI/MOPS media, RPMI/MOPS with sub-MIC antifungals, or RPMI/MOPS with sub-MIC antifungals and *C. albicans* cells at 10^6^ cells/mL. The LIPUS parameters were set to 1 MHz with a pressure of 1 W·cm^−2^ and 50% duty cycle. The cavitation probe (HCT-0320) was mounted on a ring clamp normal to the transducer/petri dish. The probe tip was set approximately 2 mm from the petri dish bottom. All measurements were repeated 3 times.

### 2.5. Growth Inhibition Assay

In vitro growth studies of *Candida* were conducted to assess the inhibitory effects of LIPUS and antifungal agents, both individually and in combination. In summary, a culture of *Candida* yeast was made in 10 mL of SD broth media and incubated with shaking at 37 °C for 48 h to ensure the stationary phase. The cells were washed twice with PBS and resuspended in RPMI/MOPS media with an optical density (O.D.) between 0.14 and 0.16 at 530 nm, measured with MicroScan Turbidity Meter (Beckman Coulter, Brea, CA, USA). A total of 1 mL of *Candida* suspensions was combined with 1 mL of AmB or MFG or an equivalent volume of RPMI/MOPS + DMSO to achieve a final O.D. that fell within 0.5 McF and the desired drug concentration [29]. A final 2 mL yeast suspension was then transferred to a 35 mm petri dish for LIPUS treatment (Figure 1). Following LIPUS exposure, 200 uL aliquots were transferred to a clear Nunclon Delta 96-well flat-bottom plate in triplicate. The plate was incubated at 37 °C in BioTek Epoch 2 (Agilent Technologies, Santa Clara, CA, USA) for 24 h. The plate was read every 30 min at 530 nm with 20 s of orbital shaking prior to each read. Experiments were repeated ≥3 times.

To rule out the thermal effects from LIPUS exposure on inhibiting *Candida* spp. growth, 1.4 mL of yeast culture samples was placed into 1.5 mL plastic tubes then subjected to heat treatment by placing said tubes on preheated heat blocks of 37 °C, 45 °C, then 5 min at 50 °C, for 5 min at each temperature. This approximated the temperature gradient produced by 1 MHz ultrasound at 1 W·cm^−2^ with a 50% duty cycle for 15 min. Control samples remained at 37 °C for 15 min. After treatment, aliquots of 200 uL were transferred to a clear 96-well flat-bottom plate in triplicate. The plate was incubated at 37 °C in Epoch 2 for 24 h. The plate was read every 30 min at 530 nm with 20 s of orbital shaking prior to each read and repeated in triplicate.

### 2.6. Model Fitting

We evaluated the effect of LIPUS on growth by obtaining 24 h of O.D. at 530 nm and plotting it against the time of incubation. We then used Prism 10.4.1 (GraphPad Prism Software LLC, Boston, MA, USA) to fit logarithmic growth models (Equation (1)) to obtain the measured OD, Y_M_ (maximum growth OD) and *k* (growth rate constant). We also calculated the time it takes to reach half Y_M_ − Y_0_ (½Y_M_) (Equation (2)).

(1) $$Y=\frac{Y_{M}\times Y_{0}}{\left(Y_{M}-Y_{0}\right)e^{-k\left(x\right)}+Y_{0}}$$

(2) $$x=\frac{ln(\frac{2Y_{M}Y_{0}}{Y_{M}-Y_{0}}-\frac{Y_{0}}{Y_{M}-Y_{0}})}{-k}$$

Equations (1) and (2). (1) Logarithmic equation; (2) time x when yeast growth half-maximum O.D. ½ (Y_M_ − Y_0_). X is the corresponding time in hours, and Y_0_ is the initial OD.

### 2.7. Time-Kill Assay

**Figure 2:**
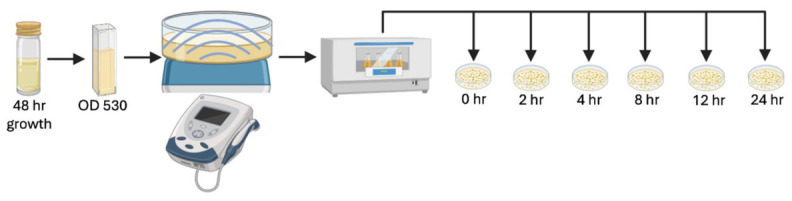
LIPUS experimental setup for time-kill assay. Arrows represent procedural next-step.

In vitro killing curves were obtained to analyze the killing effect of LIPUS and antifungals independently and in combination. Fifteen minutes post-sonication, each group was transferred to a 50 mL conical tube and incubated at 37 °C, shaking at 250 RPM for 24 h (Figure 2). At timepoints 0, 2, 4, 8, 12, and 24 h, 100 uL was removed from all treatment groups for serial dilutions, done in technical duplicates. Serial dilutions were then plated and incubated at 37 °C until colonies were countable, then colony-forming units/mL (CFUs) were calculated.

### 2.8. Adenylate Kinase Activity

Cytolysis activity was assessed through the detection of Adenylate kinase (AK) via ToxiLight™ Non-Destructive Cytotoxicity BioAssay Kit (LT17-217, Lonza, Walkersville, MD, USA) following 15 min of LIPUS exposure as described above. Previous studies have shown that antifungal treatment is strongly correlated with AK concentration in culture media, released through the disruption of the cellular membrane, activating the reagent causing luminescence, measured in relative light units (RLUs) [30,31]. The AK assay was performed according to the manufacturer’s instructions. Yeast culture preparation and sonication were identical to the growth inhibition assay and time-kill assays but differed in starting inoculum. A total of 5 × 10^6^ *C. albicans* and 5 × 10^7^ *C. glabrata* cells were treated. Samples were collected immediately post-sonication and following an 8 h incubation at 37 °C, shaking at 250 rpm. At both timepoints, 1.5 mL was removed from each group, spun down, and resuspended in 750 uL of PBS. In a white 96-well Nunc MicroWell microplate (Thermo Scientific, Waltham, MA, USA), 100 uL of suspension and 100 uL of ToxiLight reagent were added to each well in duplicate. The plate was incubated at room temperature for 45 min then luminescence was measured using BioTek Cytation 5 (Agilent Technologies, Santa Clara, CA, USA) imaging reader. Experiments were repeated ≥3 times.

### 2.9. Light Microscopy

*C. albicans* and *C. glabrata* cells were prepared using the same growth conditions as for the growth inhibition and time-kill assays. Following sonication, a 1.4 mL aliquot of the cell culture was transferred to a 1.5 mL microcentrifuge tube and centrifuged at 10,000× *g* for 5 min at room temperature. The supernatant (1.2 mL) was discarded, and the cell pellet was resuspended in the remaining 200 µL of media. A 10 µL aliquot of the cell suspension was then mixed with 10 µL of 0.4% trypan blue on a glass slide. A coverslip was applied, and the cells were visualized under a ZEISS Axio Imager.M2 microscope (Carl Zeiss Microscopy GmbH, Oberkochen, Germany) with 100× objective. Images were captured and processed using ZEN blue 3.4.91 software. Images were acquired after 5 to 7 min of incubation. Two representative fields were taken from each sample.

### 2.10. Scanning Electron Microscopy

*C. albicans* cells were fixed in 2.5% glutaraldehyde overnight then settled on silicon chips for scanning electron microscopy preparation for 20 min. The samples were washed 3 times in 0.1 M sodium cacodylate buffer and post-fixed with 1.0% osmium tetroxide/0.8% potassium ferricyanide in 0.1 M sodium cacodylate buffer for 1 h. Samples were then washed 1 time in 0.1 M sodium cacodylate followed by 2 washes in dH_2_O. Samples were then dehydrated with a graded ethanol series, critical-point-dried under CO_2_ in a Bal-Tec model CPD 030 Dryer (Balzers, Liechtenstein), mounted on aluminum studs, and sputter-coated with 15 Å of iridium in a Leica EM ACE600 high-vacuum sputter coater (Leica-Microsystems, Wetzlar, Germany) prior to viewing at 5 kV in a Hitachi SU-8000 field emission scanning electron microscope (Hitachi High-Technologies, Tokyo, Japan).

### 2.11. Statistical Analysis

Quantitative data in this study were described as the means ± standard deviations (SD) and were analyzed using GraphPad Prism 10.4.1 for MacOS (GraphPad Prism Software LLC Boston, MA, USA, www.graphpad.com). Data was tested for normality via the Shapiro–Wilk test to determine downstream parametric versus non-parametric analysis. Dunnett’s multiple comparisons test was done post hoc for ANOVA, while Dunn’s multiple comparisons test was done post hoc for the Kruskal–Wallis test. For two-way ANOVA, Šidák’s multiple comparison test was used for post hoc analysis.

Mean CFU counts (log10 CFU/mL) were plotted as a function of time for each isolate at each concentration of antifungal tested. Time-kill data was characterized as fungicidal or fungistatic as follows: fungicidal activity was defined as a ≥3 −log10 (99.9%) reduction in numbers of CFU from the starting inoculum, and fungistatic activity was defined as a <99.9% reduction in growth from the starting inoculum. Results were significant when *p*-values were <0.05.

## 3. Results

Overall, cavitation induced by the LIPUS setup was minimal, with total cavitation registering between 5 and 6% of the direct field pressure (Supplementary Table S1). No difference in cavitation was observed between degassed water, RPMI/MOPS medium, RPMI/MOPS with antifungals, and RPMI/MOPS with antifungals and yeast, indicating that the cavitation signal was below the detection threshold of the cavitation meter and did not contribute significantly to the pressure field. While SEM showed no detectable morphological differences in *C. albicans* post-LIPUS exposure compared to control (Supplementary Figure S2), light microscopy with trypan blue staining indicated increased cell membrane permeability for LIPUS-treated *C. albicans* cells, not LIPUS-treated *C. glabrata* cells (Supplementary Figure S3).

### 3.1. Minimal Inhibitory Concentration

Amphotericin B (AmB) exhibited identical MIC values for both *Candida* species (0.5 µg/mL). The MIC for *C. albicans* was 0.015 µg/mL with micafungin (MFG), while *C. glabrata* required a higher concentration (0.03 µg/mL) (Table 1).

### 3.2. Growth Inhibition

LIPUS alone has demonstrated a duration-dependent inhibitory effect. Growth curves plotted as O.D. over time are shown in Supplementary Figure S4A,B. Logarithmic growth model parameters (Y_M_, ½ Y_M_, and k) are detailed in Supplementary Table S2. For *C. albicans*, the statistical analysis showed a significant effect of LIPUS (50% duty cycle, 1 W·cm^−2^) duration on Y_M_ (*p* = 0.018), 13 min treatments significantly reducing ½ Y_M_ values (*p* < 0.001). In contrast, *C. glabrata* needed 15 min LIPUS exposure to induce a significant delay to reach ½ Y_M_. A 15 min LIPUS exposure significantly delayed the time to reach ½ Y_M_ for both species—by 2.5-fold in *C. albicans* and 1.5-fold in *C. glabrata* (Table 2). We proceeded with a 15 min LIPUS treatment for subsequent experiments.

**Figure 3:**
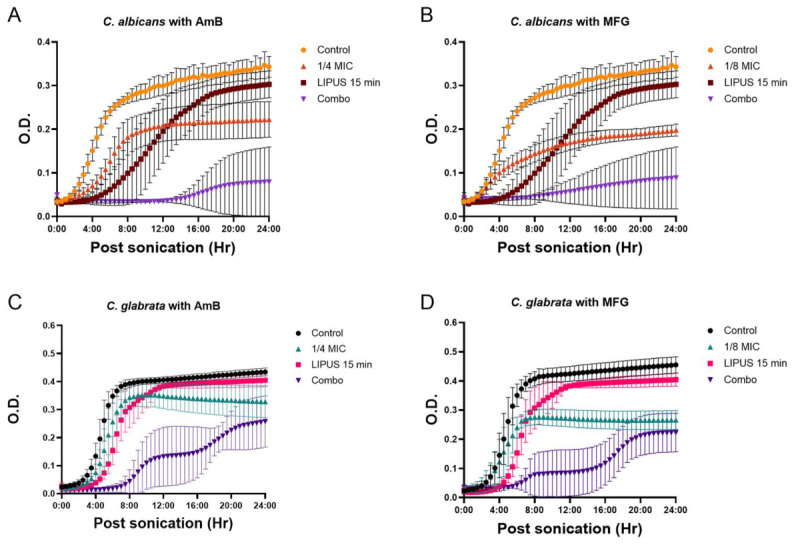
Growth inhibition curves with varying treatment combinations. *X* axis represents time post sonication treatment, while *Y* axis represents Optical Density (O.D.). *C. albicans* in combination with AmB (**A**) and MFG (**B**). *C. glabrata* in combination with AmB (**C**) and MFG (**D**). O.D. measured at 530 nm. Data are plotted as mean ± SD.

When combined with sub-MIC AmB and MFG concentrations, the LIPUS combination groups showed a marked reduction in Y_M_, *k*, and ½ Y_M_ growth parameters (Figure 3; Table 2). Notably, the inhibitory effects on *C. albicans* were significantly amplified with both antifungals; *C. glabrata* combination treatments were comparatively less affected.

For *C. albicans*, ½ Y_M_ cannot be accurately calculated for both combination treatments because most replicates had negligible growth. Similarly, for *C. glabrata*, the MFG combination group cannot be accurately calculated due to negligible growth.

**Figure 4:**
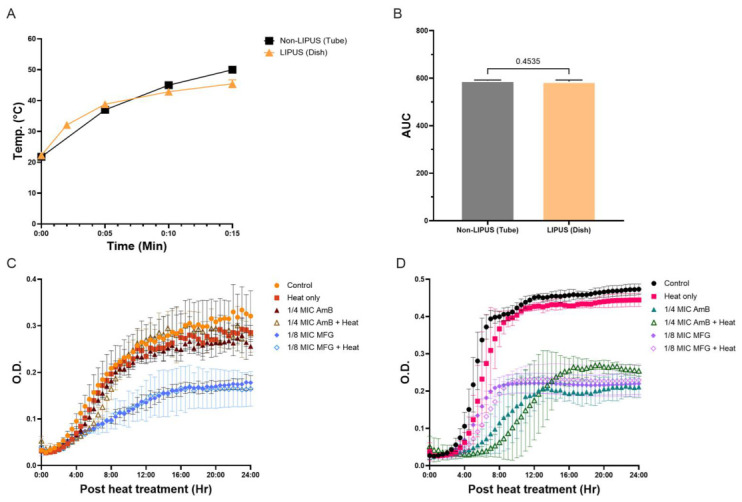
Decoupling the thermal effects of LIPUS from its effects on yeast growth. (**A**) Heat-only simulation approximated heat generated by LIPUS over course of 15 min. (**B**) Area Under the Curve (AUC) did not significantly differ between the two conditions (Welch’s *t*-test: *p* = 0.4535). (**C**,**D**) *X* axis represents time post sonication treatment, while *Y* axis represents Optical Density (O.D.). Growth curves for *C. albicans* (**C**) and *C. glabrata* (**D**) after heat treatment or after combination of heat and either antifungal also did not differ in growth kinetics. O.D. measured at 530 nm. Data plotted as mean ± SD.

Because 1 MHz LIPUS exerts a thermal effect, we assessed the impact of heat alone on the growth of yeast strains, with or without antifungals. The heat curve generated by the heat-only simulation closely approximated the curve produced by the LIPUS setup (Figure 4A). The Area Under the Curve (AUC) was not significantly different between the two conditions (AUC heat-only: 589.4 ± 1.7; LIPUS: 585.9 ± 3.7; Welch’s *t*-test: *p* = 0.4535) (Figure 4B). Heat in combination with either antifungal did not alter *C. albicans*’s growth kinetics when compared antifungal alone (Figure 4C). This holds true for *C. glabrata* as well (Figure 4D).

### 3.3. Time-Kill Assay

Time-kill assays (TKAs) were performed to evaluate the effects of LIPUS alone and in combination with antifungals on planktonic yeast cultures.

**Figure 5:**
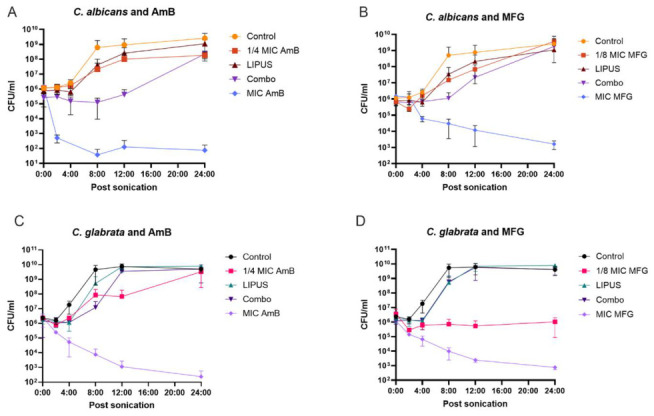
Kinetics of the antifungal effect are demonstrated via time-kill assay with different combinations of LIPUS, antifungal drugs, and *Candida* species. (**A**,**B**) *C. albicans* with AmB and MFG. (**C**,**D**) *C. glabrata* with AmB and MFG. Data plotted as mean ± SD.

The combination of LIPUS and ¼ MIC AmB exhibited fungistatic activity against *C. albicans* for up to 12 h, with CFU/mL increasing from 2.6 × 10^5^ initially to 4.2 × 10^5^ at 12 h. At this time point, the combination treatment yielded CFU counts approximately 3 log_10_ lower than those observed with LIPUS (2.5 × 10^8^) or ¼ MIC AmB (1.0 × 10^8^) alone. By 24 h, CFU levels rebounded to match control levels (Figure 5A). Similarly, the combination of ⅛ MIC MFG and LIPUS suppressed the growth of *C. albicans* for up to 8 h (1.1 × 10^6^ CFU/mL) (Figure 5B). For *C. glabrata*, all treatment groups, including LIPUS alone or with antifungals, showed ongoing growth. At 8 h, the ¼ MIC AmB + LIPUS group exhibited lower CFUs (1.2 × 10^7^) than the control (4.6 × 10^9^) (Figure 5C), but regrowth was observed 4 h later at hour 12. Notably, ⅛ MIC MFG alone produced fungistatic effects, but this was counteracted when LIPUS was applied (Figure 5D).

**Figure 6:**
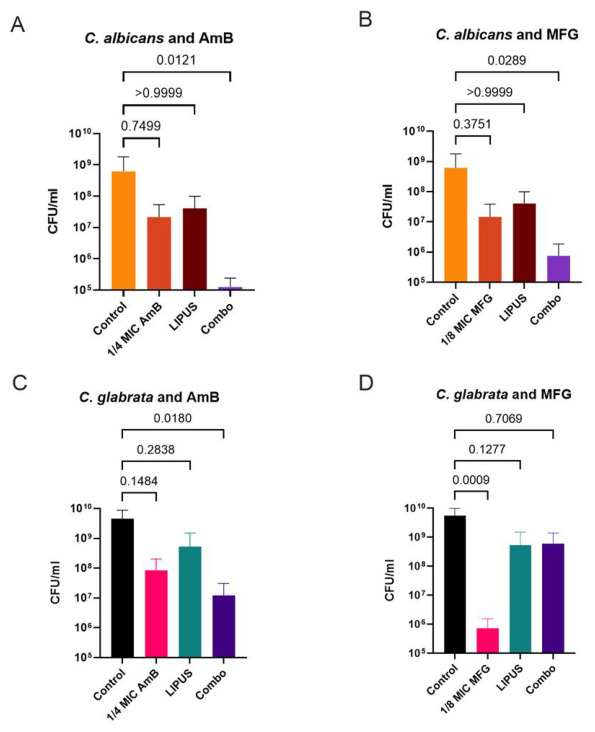
CFU/mL of AmB at ¼ MIC or MFG at ⅛ MIC with and without LIPUS at hour 8 post-sonication. Data presented as mean ± SD. *p*-values were calculated using the Kruskal–Wallis test for each *Candida* species with each antifungal agent. Post hoc comparisons were performed using Dunn’s test, with significant differences indicated on the graph. For *C. albicans*, the Kruskal–Wallis test yielded *p* = 0.019 for AmB treatment groups (**A**) and *p* = 0.044 for MFG treatment groups (**B**). For *C. glabrata*, the Kruskal–Wallis test yielded *p* = 0.013 for AmB treatment groups (**C**) and *p* = 0.004 for MFG treatment groups (**D**).

The quantitative analysis of CFU/mL at 8 h for all *Candida* species and antifungal combinations is presented in Figure 6. CFU/mL values were analyzed using a Kruskal–Wallis (KW) test with Dunn’s post hoc analysis. For *C. albicans*, treatment groups differed significantly among AmB groups (KW: *p* = 0.019; Figure 6A) and MFG groups (KW: *p* = 0.044; Figure 6B). Post hoc analysis showed that combination treatment of LIPUS with ¼ AmB (*p* = 0.012) and ⅛ MFG (*p* = 0.029) produced significantly lower counts than control. For *C. glabrata*, significant differences among treatment groups were observed for both antifungals (KW: AmB, *p* = 0.0126; KW: MFG, *p* = 0.0037; Figure 6C,D). However, post hoc analysis showed significance only between control and the ¼ AmB combination treatment (*p* = 0.018), whereas the ⅛ MIC MFG combination did not differ significantly from the control (*p* = 0.71). Notably, ⅛ MFG alone was significantly lower than the control (*p* < 0.001).

### 3.4. Adenylate Kinase Release Differs Between Candida Species

Adenylate kinase (AK) is an intracellular enzyme that catalyzes the phosphoryl transfer between two adenosine diphosphate molecules to yield adenosine triphosphate and adenosine monophosphate. AK release is correlated with compromised cell membrane integrity, which allows the enzyme to translocate into the extracellular environment. When released into the surrounding medium, AK activity can be detected using a commercially available luminescent assay in which AK-generated ATP activates luciferase, producing luminescence measured in relative light units (RLU) [32]. We utilized the AK assay to assess the effects of ultrasound alone and in combination with AmB on yeast cell membrane integrity.

**Figure 7:**
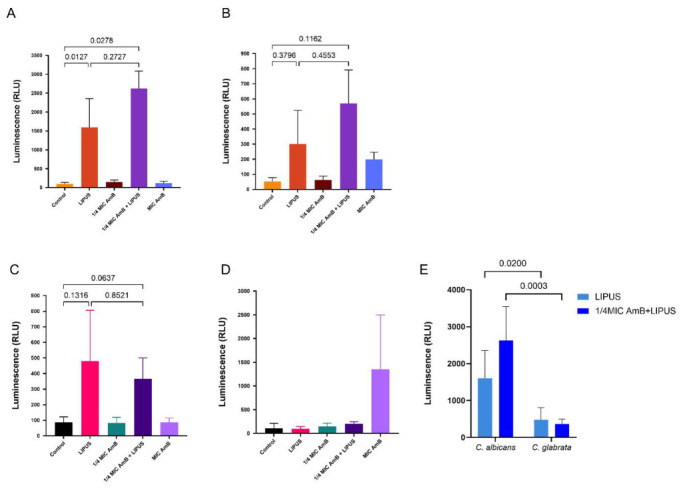
Adenylate kinase release after LIPUS treatment in *Candida* spp. *C. albicans* at 0 h and 8 h (**A**,**B**) and *C. glabrata* at 0 h and 8 h (**C**,**D**). *p*-values were calculated using Brown–Forsythe ANOVA to compare treatment groups for *C. albicans* at 0 h (*p* = 0.001) and 8 h (*p* = 0.034), and for *C. glabrata* at 0 h (*p* = 0.024) and 8 h (*p* = 0.12). Side-by-side comparison of 0 h AK levels between the two *Candida* species (**E**) using two-way ANOVA with Šidák’s multiple comparison test for post hoc analysis.

**Figure 8:**
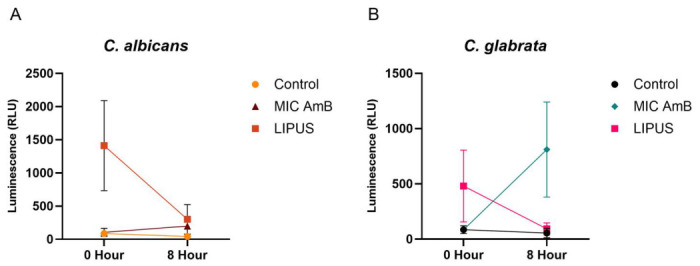
Adenylate kinase level. *X* axis represents time post sonication treatment, while *Y* axis represents Relative Luminescence Unit (RLU). Changes over time among different treatment groups for *C. albicans* (**A**) and *C. glabrata* (**B**).

We first examined AK release in *C. albicans*. Immediately post-sonication (Time 0), we observed significant differences in AK release among the various *C. albicans* treatment groups via one-way ANOVA (*p* = 0.001) (Figure 7A). Post hoc comparison revealed that both LIPUS-only (RLU 1608.5 ± 597.5, *p* = 0.013) and the combination LIPUS + ¼ MIC AmB group (RLU 2448.7 ± 1036.1, *p* = 0.028) exhibited significantly higher AK release compared to yeast control cells (RLU 85.9 ± 25.5). After 8 h of incubation, treatment groups still showed statistically different AK levels (one-way ANOVA, *p* = 0.034) (Figure 7B). LIPUS (RLU 300.8 ± 224) and ¼ MIC AmB + LIPUS (RLU 570.3 ± 221.7) still exhibited the highest RLUs of all treatment groups, though these values had decreased from Time 0. However, post hoc analysis at 8 h showed no significant differences between these treatment groups and the yeast-only control (Figure 7B). The AmB MIC group showed an RLU of 199.5 ± 46.7, while untreated yeast cells had an RLU of 42.0 ± 22.3. Figure 8A depicts *C. albicans* AK level changes over 8 h of incubation for different treatment groups.

For *C. glabrata*, the pattern differed. Immediately post-sonication (Time 0), treatment groups showed overall statistical differences (one-way ANOVA, *p* = 0.024) (Figure 7C). However, neither LIPUS alone (480.1 ± 325.3 RLU, *p* = 0.13) nor LIPUS combined with ¼ MIC AmB (365.0 ± 134.5 RLU, *p* = 0.064) caused significantly more AK release than untreated controls (86.0 ± 35.8 RLU), indicating less membrane leakage. At Time 8, *C. glabrata* treatment groups showed no statistically significant differences (*p* = 0.12) (Figure 7D). After 8 h, there were no significant differences among any *C. glabrata* treatment groups (*p* = 0.12) (Figure 7D). The time course of AK release for all *C. glabrata* treatment groups is shown in Figure 8B.

For Figure 7E, we compared AK release at Time 0 between *C. albicans* and *C. glabrata* using two-way ANOVA. We examined whether AK levels differed by species type and whether adding ¼ MIC AmB to LIPUS altered this response. Species type had a highly significant effect on AK release (*p* < 0.0001), but adding AmB did not (*p* = 0.14), and there was no significant interaction between these factors (*p* = 0.069). Šidák’s post hoc analysis confirmed that *C. albicans* released significantly more AK than *C. glabrata* in response to both LIPUS alone (*p* = 0.02) and LIPUS combined with ¼ MIC AmB (*p* = 0.0003). This strain-specific difference in AK release is consistent with our time-kill assay data, which showed that *C. glabrata* was less susceptible to LIPUS treatment (Figure 8).

## 4. Discussion

Our findings demonstrate that LIPUS enhances transient but marked inhibitory effects of sub-MIC AmB and MFG against *C. albicans*, but not *C. glabrata*. Our *C. albicans* results are consistent with prior reports that LIPUS can increase membrane permeability and drug susceptibility [15,22,24]. Yang et al. previously reported synergistic effects of LIPUS with AmB using nanoparticles against *C. albicans*, but their study used a lower frequency (42 kHz) and did not evaluate time-dependent fungicidal activity [24]. In contrast, our work utilizes a clinically relevant 1 MHz frequency and includes 24 h time-kill curves and AK assays, providing a dynamic view of antifungal activity and cell integrity over time.

While prior studies have focused on bacterial models or yeast species under optimized drug conditions [24,26], we uniquely explore LIPUS efficacy at sub-MIC levels, mimicking clinically relevant tissue concentrations where drug penetration can be limited [27,28]. The limited effect observed in *C. glabrata* in response to LIPUS and its unexpected response to sub-MIC MFG post-sonication are notable. We postulate that *C. glabrata*, with its well-documented adaptive capacity, such as its rapid development of echinocandin resistance via FKS mutations [8], was able to respond to LIPUS’ downstream biological consequences better than *C. albicans*. While we only tested one strain of each *Candida* species, our findings reveal that there is potential for LIPUS-based adjunct therapies to be counterproductive with certain *Candida* strains. Thus, we emphasize the importance of tailoring LIPUS-based adjunct therapies to the pathogen and the antifungal class, and support further investigation in in vivo models.

Growth inhibition in our experiments was dependent on sonication duration and differed between species. Growth inhibition began at 15 min post-LIPUS exposure, with differences in recovery kinetics between *C. albicans* (approximately 7 h to reach ½ Y_M_) and *C. glabrata* (approximately 3 h to reach ½ Y_M_) (Supplementary Table S2). Combining LIPUS with sub-MIC AmB and MFG made *C. albicans* more susceptible to treatment when compared to *C. glabrata*.

To probe cell integrity, we used the AK assay, which Krysan et al. first described as a surrogate for yeast cell lysis when exposed to caspofungin, which is structurally similar to MFG [32]. We observed a greater leakage of AK from *C. albicans* following LIPUS compared to *C. glabrata*. In concurrence, our light microscopy showed that only the *C. albicans* strain was permeable to trypan blue stain post-sonication, while the *C. glabrata* strain did not exhibit any staining (Supplementary Figure S3). One speculation of the difference in AK leakage may be due to the larger surface of *C. albicans* (1 to 36 times) compared to *C. glabrata* [33,34] resulting in greater membrane instability when exposed to acoustic radiation forces of LIPUS. Although AK release provides an indirect readout consistent with membrane destabilization, it does not distinguish transient permeability from irreversible lysis. Future studies would therefore benefit from complementary viability and membrane-integrity assays, such as live/dead staining with propidium iodide or SYTOX Green, to better resolve the extent and kinetics of ultrasound-induced permeability changes.

We did not observe a measurable presence of cavitation by our instrument when LIPUS was applied in degassed water, growth media with or without antifungals and cells (Supplementary Table S1), suggesting that downstream effects were mainly caused by mechanical force, rather than cavitation, which were the primary contributors to growth inhibition. While our instrument could not pick up cavitary forces, it does not completely rule out the presence of micro-cavitation surrounding the cells, below our instrument’s level of detection. On the other hand, SEM images of *C. albicans* after 15 min of LIPUS did not show cell-wall damage (Supplementary Figure S2B). Together, these findings are more consistent with a predominantly non-cavitation mechanism than with cavitation-mediated cell-wall disruption.

In comparison with prior studies, Yang et al. saw an immediate ~40% reduction in CFU/mL count with ½ MIC AmB by itself [24]. However, we did not see an immediate reduction in CFU/mL count in ¼ MIC AmB nor AmB at MIC treatments for both *Candida* species. Notably, Yang and colleagues showed that the survival rate of *C. albicans* was unaffected using LIPUS at 42 KHz alone, which is consistent with our results at 1 MHz. Rocha et al. reported an increase in CFU/mL after 10–15 min of continuous sonication for *C. albicans* [26]. They combined LIPUS with fluconazole at 12 ug/mL with *C. albicans* (ATCC 10231) and demonstrated an increase in killing (i.e., ~66% reduction in CFU/mL) starting at 5 min, but 12 ug/mL was equivalent to 24× MIC, which is clinically unachievable. Moreover, the study lacked long-term (i.e., 24 h) follow-up on the fungicidal effects.

From our time-kill assays, antifungal effects are variable at different concentrations with different organisms. AmB and MFG are fungicidal at MIC levels, but both drugs were not fungistatic at sub-MIC levels, except for *C. glabrata* with ⅛ MIC MFG. The ¼ MIC AmB + LIPUS treatment groups for both *Candida* species showed statistically significant delays in growth by 8 h and specifically 12 h for *C. albicans* post-sonication. We hypothesize the antifungal’s mechanism of action on the cell membrane (AmB) [35] or downstream effect on cell-wall synthesis (MFG) [36] may contribute to the observed differences. However, by 24 h, there were no differences in CFU/mL.

Unexpectedly, LIPUS combined with ⅛ MIC MFG promoted *C. glabrata* growth rather than inhibiting it (Figure 5D), indicating that ultrasound at sub-MIC drug concentrations may exhibit strain-dependent effects that could be counterproductive if the wrong combination is selected. We speculate that this observation can partially be explained by how the two *Candida* species respond to environmental stress. In our experimental setup, cells experienced multiple stressors, including heating, cell-wall stress (from antifungal exposure), and direct mechanical stimulation from ultrasound. Heat and cell-wall stress are known to alter gene expression in both *C. glabrata* and *C. albicans* in ways that have been linked to antifungal resistance phenotypes [37,38,39]. Moreover, susceptibility patterns can differ across *Candida* species as a function of both antifungal class and temperature. For example, Odds reported that *C. glabrata* exhibited greater tolerance to azoles at 37 °C than at 25 °C, whereas *C. albicans* showed the opposite trend [7]. However, we demonstrated that heat alone or in combination with antifungals does not change the overall growth curve for both yeast species (Figure 4C,D), suggesting that temperature is unlikely to be the primary driver we observed for the growth enhancement.

Instead, the differential outcome may reflect *C. glabrata*’s particularly robust stress-response network, which can promote survival under hostile conditions. For example, *C. glabrata* exhibits enhanced survival during macrophage phagocytosis through efficient mitigation of reactive oxygen species (ROS), mediated in part by the transcriptional regulators MSN2 and MSN4 [40,41,42], mechanisms not mirrored in *C. albicans*. Consistent with this, Cuéllar-Cruz et al. showed that *C. glabrata* tolerates higher levels of H_2_O_2_ than *C. albicans* and *S. cerevisiae*, and that this phenotype depends on the catalase gene *CTA1* [43]. On the other hand, LIPUS has been reported to induce ROS generation [24], and therefore we infer that *C. glabrata* may be comparatively less susceptible to ultrasound-associated oxidative stress. Under sub-MIC echinocandin pressure, this stress tolerance could allow *C. glabrata* not only to persist but potentially to shift into a compensatory or adaptive state that manifests as increased growth. Lastly, to date, the mechanotransductive sensitivity of either species to LIPUS, alone or in combination with antifungals, remains insufficiently characterized. In addition, through an extensive literature search, we have yet to find how mechanical stimulation affects *C. albican*’s virulence factors such as hyphal formation. While there is literature on *Candida* biofilm inactivation [25,44], there is sparse literature on biofilm production post-sonication. These areas warrant further targeted investigation.

There are several study limitations that provide motivation for future work. First, because this was a proof-of-concept study, we evaluated only a single strain of each *Candida* species. Follow-up studies should include a broader panel of clinical isolates, as isolates can vary substantially in growth kinetics, virulence-associated traits, and antifungal susceptibilities. Second, testing a wider range of sub-MIC antifungal concentrations would better define the conditions under which LIPUS may be beneficial, neutral, or counterproductive. Although the concentrations employed here were selected based on published reports of limited antifungal penetration at sites of infection, more comprehensive concentration-response experiments will be an essential next step in validating and extending these findings. Third, our model setup indicates sonicating our samples through a small plastic petri dish, which introduces unavoidable vibrations, a limitation that is a result of LIPUS equipment, and the introduction of heat during LIPUS, which cannot be minimized, can contribute to yeast cell stress responses [39]. Of note, LIPUS at 1 MHz will inherently induce more thermal effects in comparison to 42 KHz, which was used by Yang et al. to effectively reduce CFU in *C. albicans* [24]. Finally, *Candida* pathogenesis involves virulence factors, most notably biofilm formation and, in some species such as *C. albicans*, filamentation, that promote tissue invasion and persistence on indwelling devices (e.g., catheters and implants) [45]. Although prior studies have examined LIPUS-mediated disruption of established biofilms in *C. albicans*, the effects of LIPUS on biofilm formation and hyphal formation remain largely uncharacterized. Accordingly, evaluating LIPUS in biofilm and filamentation models, particularly in the presence of sub-MIC antifungals, represents an important and clinically relevant next step. All in all, further in vitro and in vivo studies are needed to elucidate how LIPUS interacts with different *Candida* strains and species with antifungal exposures across diverse isolates and infection-relevant phenotypes.

## 5. Conclusions

This study demonstrates that LIPUS can disrupt the cell membrane of *C. albicans* ATCC 10231 and *C. glabrata* ATCC 15126, as evidenced by AK release. While LIPUS alone or in combination with sub-MIC antifungal concentrations did not produce sustained additive antifungal effects, a transient fungistatic response was observed in *C. albicans*, particularly when paired with AmB. In contrast, *C. glabrata* demonstrated greater resilience, with LIPUS exposure sometimes diminishing antifungal efficacy, highlighting a strain-specific variability in response to ultrasound-mediated stress. Together, these results suggest that the therapeutic utility of LIPUS as an adjunct to antifungal therapy may be conditional, depending on both the infecting *Candida* strains and the antifungal class used. As proof of concept, this work provides a foundation for evaluating LIPUS as a noninvasive, localized adjunct to antifungal treatment. However, because fungal organisms can respond differently to sonication, defining organism-specific ultrasound parameters and demonstrating in vivo efficacy will be essential next steps in determining clinical utility.

## Figures and Tables

**Table 1:** MIC values for *Candida* species.

Species	AmB MIC (µg/mL)	MFG MIC (µg/mL)
*C. albicans* ATCC 10231	0.5	0.015
*C. glabrata* ATCC 15126	0.5	0.03

**Table 2:** Growth parameters for *C. albicans* and *C. glabrata* treated with antifungals and/or LIPUS. Welch’s ANOVA with Dunnett’s multiple comparison test was performed. *, **, ***, and **** indicate *p* < 0.05, 0.01, 0.001, and 0.0001, respectively. & indicates minimal or no growth observed.

	*C. albicans*			*C. glabrata*		
Treatment	Y_M_ (O.D.)	*k*	½ YM (h)	Y_M_ (O.D.)	*k*	½ Y_M_ (h)
Control	0.33	0.49	4.52	0.44	0.88	5.15
15 min LIPUS	0.29	0.35	11.53 *	0.38	0.86	7.63 *
¼ MIC AmB	0.22	0.48	4.80	0.34	1.31 *	5.12
¼ MIC AmB + LIPUS	0.14	0.18 **	22.43 &****	0.19	0.46	7.30 ***
⅛ MIC MFG	0.19	0.32	3.05	0.27	1.14	4.24
⅛ MIC MFG + LIPUS	0.089 ****	0.074 **	10.89 &**	0.26 &	0.16 ****	3.01 &**

## Data Availability

The data presented within this article are available from the corresponding author on request.

## Supplementary Materials

The following supporting information can be downloaded at: https://www.mdpi.com/article/10.3390/jof12060399/s1, Figure S1: Force balance readings to calibrate Mettler Sonicator 740 units; Figure S2: Scanning Electron Microscopy images. *C. albicans* without sonication (A) and *C. albicans* following 15 minutes of LIPUS treatment at 50% duty cycle, 1 W·cm^−2^ (B); Figure S3: Light microscopy images. *C. albicans* images (A–D) and *C. glabarata* images (E–H) are presented with trypan blue. *C. albicans* post sonication (C,D) exhibited more trypan blue dye leakage into cell when compared to control (A,B). On the other hand, there was no trypan blue dye leakage into *C. glabrata* cells post sonication (G,H) when compared to its control (E,F); Figure S4: Growth inhibition growth curves with varying durations of LIPUS treatment times. Time duration ranged from 1 min to 13 min. Duration of LIPUS treatment shifts growth curve to the right for both *C. albicans* (A) and *C. glabrata* (B). Data plotted as mean ± SD; Table S1: Direct field and cavitation readings. Readings done at 1 W·cm^−2^ with total cavitation calculated as the sum of the stable and transient cavitation pressure; Table S2: Sonication parameters at different sonication durations. Sonication was done at 50% duty cycle, 1 W·cm^−2^. The *p*-values calculated via Welch’s ANOVA with Dunnett’s multiple comparison test. * *p* < 0.05, ** *p* < 0.01, *** *p* < 0.001, **** *p* < 0.0001.

## Abbreviations

The following abbreviations are used in this manuscript:

LIPUS	Low-intensity pulsed ultrasound
MIC	Minimal inhibitory concentration
AmB	Amphotericin B
MFG	Micafungin
AK	Adenylate Kinase
AIDS	Acquired Immunodeficiency Syndrome
IFI	Invasive fungal infections
TUS	Therapeutic Ultrasound
MB	Microbubbles
SD	Sabaroud dextrose
RPMI	Roswell Park Memorial Institute
MOPS	3-(N-morpholino) propanesulfonic acid
McF	McFarland
OD	Optical density
YM	Maximum growth OD
k	Growth rate constant
CFU	Colony-forming units
RLU	Relative Light Unit
TKA	Time-kill assay
KW	Kruskal–Wallis
ROS	Reactive oxygen species
