# Supplementary material for: Differential Effects of Low-Intensity Pulsed Ultrasound and Antifungals on Candida albicans and Candida glabrata: Implications for Drug Efficacy
Source: J Fungi (Basel). 2026 May 30;12(6):399. doi: 10.3390/jof12060399 (PMC13300981; doi:10.3390/jof12060399)
Supplement: Supplementary file 1 [file jof-12-00399-s001.zip › jof-4185703-supplementary.pdf]

Supplemental Materials

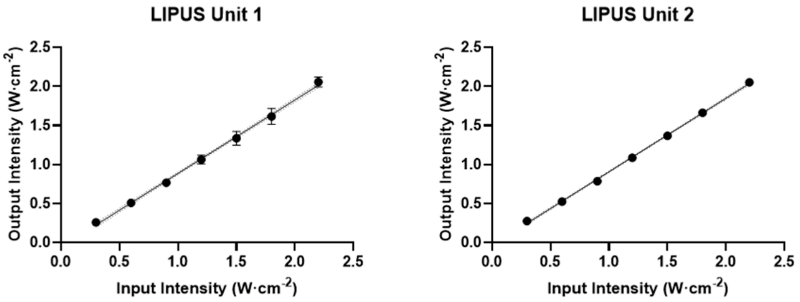

Figure S1: Force balance readings to calibrate Mettler Sonicator 740 units.

Table S1: Direct field and cavitation readings. Readings done at 1 W·cm^{-2} with total cavitation calculated as the sum of the stable and transient cavitation pressure.

Group	Degassed H_2O	RPMI/MOPS	RPMI/MOPS +1/4MIC AmB	RPMI/MOPS +1/8MIC MFG	RPMI/MOPS +1/4MIC AmB+Cells	RPMI/MOPS +1/8MIC MFG+Cells
	Mean±SD	Mean±SD	Mean±SD	Mean±SD	Mean±SD	Mean±SD
Direct field pressure	40±4.4 kPa	39±3.6 kPa	37.7±2.1 kPa	39.7±2.5 kPa	39.3±2.0 kPa	37.7±3.8 kPa
Stable cavitation pressure	1.0±0.0 kPa	1.0±0.0 kPa	1.3±0.5 kPa	1±0 kPa	1.3±0.6 kPa	1.3±0.6 kPa
Transient cavitation pressure	0.6±0.5 kPa	1.0±0.0 kPa	1±0 kPa	1±0 kPa	1.0±0.0 kPa	1±0.0 kPa
Total cavitation pressure	1.7±0.6 kPa	2±0.4 kPa	2.3±0.6 kPa	2±0 kPa	2.3±0.6 kPa	2.0±0.0 kPa
Percent total cavitation	4.3±1.8 %	5.1±0.5 %	6.2±1.2%	5.01±0.3%	5.9±1.4%	5.3±0.5%

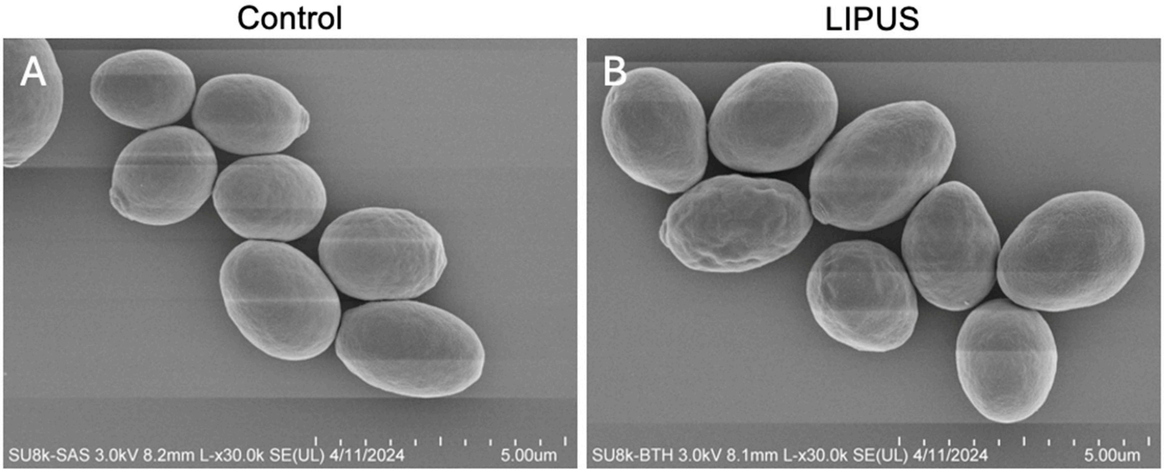

Figure *S2*: Scanning Electron Microscopy images. *C. albicans* without sonication (A) and *C. albicans* following 15 minutes of LIPUS treatment at 50% duty cycle, 1 W·cm^{-2} (B).

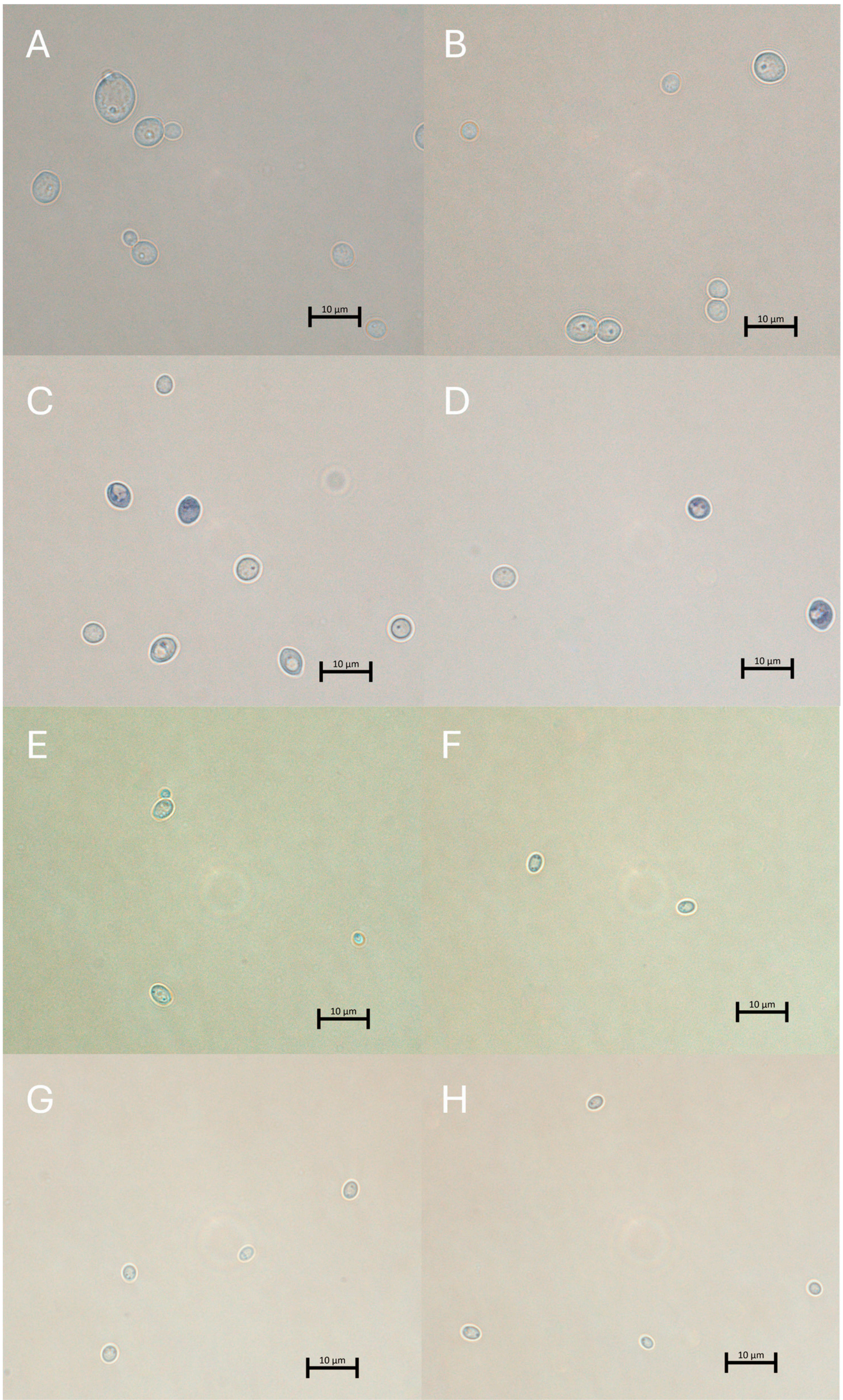

Figure S3. Light microscopy images. *C. albicans* images (A-D) and *C. glabarata* images (E-H) are presented with trypan blue. *C. albicans* post sonication (C,D) exhibited more trypan blue dye leakage into cell when

compared to control (A,B). On the other hand, there was no trypan blue dye leakage into *C. glabrata* cells post sonication (G,H) when compared to its control (E,F).

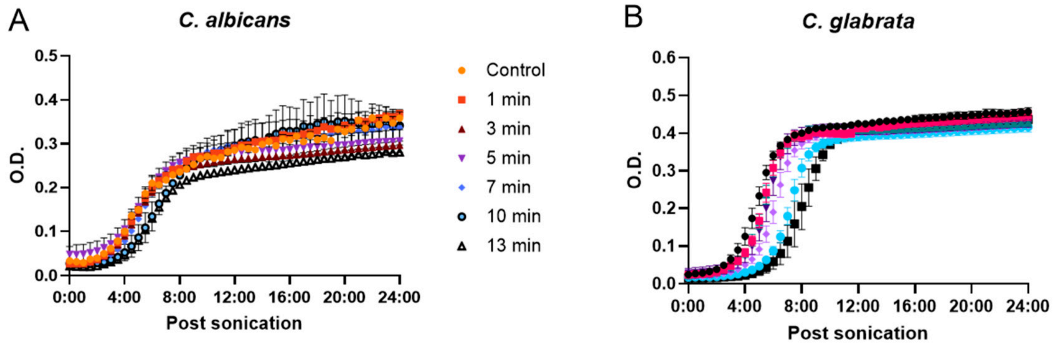

Figure S4: Growth inhibition growth curves with varying durations of LIPUS treatment times. Time duration ranged from 1 min to 13 min. Duration of LIPUS treatment shifts growth curve to the right for both *C. albicans* (A) and *C. glabrata* (B). Data plotted as mean ± SD.

Table S2: Sonication parameters at different sonication durations. Sonication was done at 50% duty cycle, 1 W·cm^{-2}. The p-values calculated via Welch's ANOVA with Dunnett's multiple comparison test. * p<0.05, ** p<0.01, *** p<0.001, **** p<0.0001

C. albicans	**Control**	**1 min**	**3 min**	**5 min**	**7 min**	**10 min**	**13 min**	**p-value**
**Y_M (O.D.)**	0.33	0.31	0.27**	0.29	0.30	0.31	0.26***	0.018
**k**	0.49	0.45	0.59	0.56	0.58	0.55	0.59	0.055
**½ Y_M (Hour)**	4.52	5.29	4.86	4.55	5.44	6.07	6.19**	0.0002
C. glabrata	**Control**	**1 min**	**3 min**	**5 min**	**7 min**	**10 min**	**13 min**	**p-value**
**Y_M (O.D.)**	0.44	0.43	0.42	0.42	0.41	0.40	0.42	0.67
**k**	0.88	1.01	1.13	1.12	1.13	1.07	1.00	0.40
**½ Y_M (Hour)**	5.16	5.26	5.22	5.41	5.79	6.00	8.00	0.09
